# Supplementary material for: Single Cell Transcriptomic and Chromatin Profiles Suggest Layer Vb Is the Only Layer With Shared Excitatory Cell Types in the Medial and Lateral Entorhinal Cortex
Source: Front Neural Circuits. 2022 Jan 26;15:806154. doi: 10.3389/fncir.2021.806154 (PMC8826650; doi:10.3389/fncir.2021.806154)
Supplement: Supplementary file 14 [file Data_Sheet_1.ZIP › Exc_vs_Inh_Motifs/homerResults/motif6.similar.html]

motif6

## Information for motif6

A
C
G
T
C
T
G
A
C
G
T
A
A
G
T
C
C
G
T
A
A
G
C
T
A
C
G
T
C
G
A
T
  
Reverse Opposite:  

C
G
T
A
C
G
T
A
C
T
G
A
A
C
G
T
C
T
A
G
A
C
G
T
A
G
C
T
C
G
T
A
  

|  |  |
| --- | --- |
| p-value: | 1e-314 |
| log p-value: | -7.248e+02 |
| Information Content per bp: | 1.870 |
| Number of Target Sequences with motif | 10670.0 |
| Percentage of Target Sequences with motif | 25.29% |
| Number of Background Sequences with motif | 1943.3 |
| Percentage of Background Sequences with motif | 17.88% |
| Average Position of motif in Targets | 336.5 +/- 246.5bp |
| Average Position of motif in Background | 317.4 +/- 279.4bp |
| Strand Bias (log2 ratio + to - strand density) | -0.0 |
| Multiplicity (# of sites on avg that occur together) | 1.23 |
| Motif File: | file (matrix) reverse opposite |

### Similar de novo motifs found

|  |  |  |  |  |  |  |  |
| --- | --- | --- | --- | --- | --- | --- | --- |
| Rank | Match Score | Redundant Motif | P-value | log P-value | % of Targets | % of Background | Motif file |
| 1 | 0.669 | A C G T A G C T A C T G C G T A A G T C C G T A A C G T A G C T A C G T C G T A | 1e-167 | -385.315938 | 59.51% | 52.83% | motif file (matrix) |
| 2 | 0.615 | G T A C C A T G A C T G C G A T T C G A G A T C G C T A A C G T C G A T G A C T G C T A G A T C | 1e-50 | -116.702997 | 0.30% | 0.06% | motif file (matrix) |
